# Efficacy of a large language model in classifying branch-duct intraductal papillary mucinous neoplasms

**DOI:** 10.1007/s00261-025-05062-z

**Published:** 2025-06-11

**Authors:** Mai Sato, Koichiro Yasaka, Shimon Abe, Joji Kurashima, Yusuke Asari, Shigeru Kiryu, Osamu Abe

**Affiliations:** 1 The University of Tokyo, Tokyo, Japan; 2 International University of Health and Welfare, Ōtawara, Japan

**Keywords:** Pancreas, Intraductal papillary mucinous neoplasms, MRI, Radiology report, Deep learning, Natural language processing

## Abstract

**Objectives:**

Appropriate categorization based on magnetic resonance imaging (MRI) findings is important for managing intraductal papillary mucinous neoplasms (IPMNs). In this study, a large language model (LLM) that classifies IPMNs based on MRI findings was developed, and its performance was compared with that of less experienced human readers.

**Methods:**

The medical image management and processing systems of our hospital were searched to identify MRI reports of branch-duct IPMNs (BD-IPMNs). They were assigned to the training, validation, and testing datasets in chronological order. The model was trained on the training dataset, and the best-performing model on the validation dataset was evaluated on the test dataset. Furthermore, two radiology residents (Readers 1 and 2) and an intern (Reader 3) manually sorted the reports in the test dataset. The accuracy, sensitivity, and time required for categorizing were compared between the model and readers.

**Results:**

The accuracy of the fine-tuned LLM for the test dataset was 0.966, which was comparable to that of Readers 1 and 2 (0.931–0.972) and significantly better than that of Reader 3 (0.907). The fine-tuned LLM had an area under the receiver operating characteristic curve of 0.982 for the classification of cyst diameter ≥ 10 mm, which was significantly superior to that of Reader 3 (0.944). Furthermore, the fine-tuned LLM (25 s) completed the test dataset faster than the readers (1,887–2,646 s).

**Conclusion:**

The fine-tuned LLM classified BD-IPMNs based on MRI findings with comparable performance to that of radiology residents and significantly reduced the time required.

**Supplementary Information:**

The online version contains supplementary material available at 10.1007/s00261-025-05062-z.

## Introduction

Intraductal papillary mucinous neoplasm (IPMN) is an exocrine tumor of the pancreas composed of mucin-producing epithelial cells and is a common disease, accounting for 1% of all pancreatic tumors and 25% of all pancreatic cystic tumors [1]. IPMNs are classified into three subtypes according to the involvement of the pancreatic ducts: main duct IPMNs, branch-duct IPMNs (BD-IPMNs), and mixed-type IPMNs [2]. IPMNs are considered precancerous lesions of pancreatic cancer [1], and adenoma-carcinoma sequence is observed [1]. It is reported that the time from low-grade dysplasia to the development of invasive cancer is about 4–6 years [1], and it is reported that the 5-year survival rate of non-malignant IPMNs is from 77 to 100%, and malignant IPMNs from 22 to 62% [3]. Furthermore, other comorbid cancers arise from a pancreatic duct other than the one in which IPMNs exist [4]. Therefore, appropriate follow-up of IPMNs is important for the early detection of cancer and therapeutic intervention.

BD-IPMN is the most common IPMN subtype, and the Revised International Consensus Fukuoka Guidelines (2017) provide an algorithm for selecting treatment strategies and follow-up intervals for BD-IPMNs. These guidelines define *high-risk stigmata (HRS)* and *worrisome features (WF)* as signs of suspected malignancy that should be considered for close examination [5]. It has been reported that 70% of patients with HRS and 30% of patients with WF have malignancies, and a stepwise increase in risk with the number of WF cases [6]. Therefore, following the guidelines is important for intervention and management. Thus, radiologists should write reports according to the guidelines. However, some familiarity and experience may be required to make these decisions in daily practice. Therefore, tools that can assist less experienced human readers are essential.

Applications of deep learning in the field of radiology have gained wide attention since the mid-2010s [7]. This technique can be applied not only to image-based tasks, including image processing [8–10] and imaging diagnosis [11–13], but also to natural language processing [14–18]. Some methods, such as ChatGPT and Bidirectional Encoder Representation from Transformers (BERT), can be used for natural language processing tasks. Although ChatGPT requires the upload of data to an Internet server, BERT can be downloaded to local computers and can be used without privacy concerns [19]. BERT has shown promising results in classifying radiology reports in several tasks [14–18]. Therefore, BERT would have a potential to classify magnetic resonance imaging (MRI) reports according to the Revised International Consensus Fukuoka Guidelines and may help radiologists who are unfamiliar with these guidelines.

In this study, a large language model (LLM) was developed to classify MRI reports of BD-IPMNs based on the guidelines, and its performance was compared with that of less experienced human readers.

## Materials and methods

This retrospective study was approved by the Institutional Review Board of our institution, which waived the requirement for obtaining written informed consent because of the retrospective nature of this study.

### Patients

**Fig. 1:**
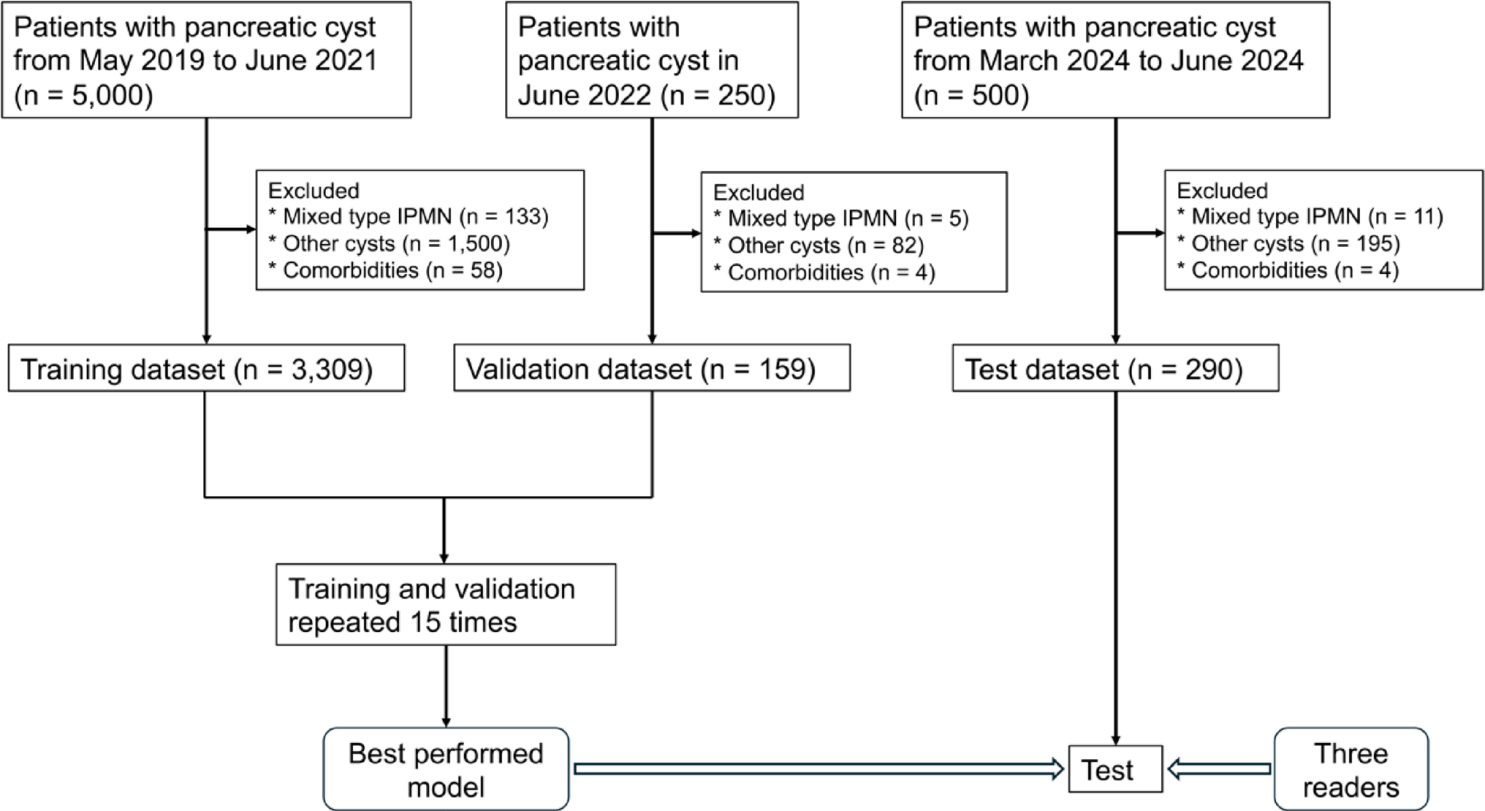
Patient inclusion and exclusion process

The medical image management and processing systems of our hospital were searched to identify appropriate cases, and radiology reports from MRI examinations of the upper abdomen were gathered; these reports were then assigned to the training, validation, and testing datasets (Fig. 1). The training dataset comprised 5,000 cases of upper abdominal MRI reports with pancreatic cysts obtained between May 2019 and June 2021; the validation dataset comprised 250 cases of upper abdominal MRI reports with pancreatic cysts obtained in June 2022; and the test dataset comprised 500 cases of upper abdominal MRI reports with pancreatic cysts obtained between March 2024 and June 2024. We selected cases that were morphologically consistent with BD-IPMN. MRI reports that did not include pancreatic cysts, patients with nonspecific cysts not determined as BD-IPMNs, main duct IPMNs without pancreatic cysts, and mixed-type IPMNs were excluded from all datasets. Main-duct and mixed-type IPMNs were excluded because the Revised International Consensus Fukuoka Guidelines (2017) [5], which we used as the basis for classification, provide recommendations for follow-up intervals and intervention only for BD-IPMNs. MRI examination included the following sequences; 2D-magnetic resonance cholangiopancreatography, 3D-mangetic resonance cholangiopancreatography, true fast imaging with steady precession images, single shot fast spin echo T2-weighted images, diffusion weighted images, 3D fat-suppressed T1-weighted images. From the test dataset, patients included in the training dataset were excluded. Reports were obtained in CSV format. In our institution, there is no dedicated report template for IPMNs. All reports were written in Japanese, and words such as HRS and WF were removed from the reports.

### Reference standard

Each report was classified into five groups according to the Revised International Consensus Fukuoka Guidelines (2017) [5] by a radiology resident with 1 year of diagnostic imaging experience. The groups were as follows:

*Group 0: BD-IPMNs difficult to classify based on the Revised International Consensus Fukuoka Guidelines (e.g., no description of the maximum cyst diameter).

*Group 1: BD-IPMNs with cyst diameters of < 10 mm.

*Group 2: BD-IPMNs with cyst diameters between 10 and 19 mm.

*Group 3: BD-IPMNs with cyst diameters between 20 and 29 mm.

*Group 4: BD-IPMNs with WF (cysts ≥ 3 cm, enhancing mural nodules ≤ 5 mm, thickened or enhancing cyst walls, main pancreatic duct diameter of 5–9 mm, abrupt change in the caliber of the pancreatic duct with distal pancreatic atrophy, lymphadenopathy, elevated serum CA19-9 levels, and a cyst growth rate of ≥ 5 mm over 2 years) or HRS (obstructive jaundice in a patient with a cystic lesion in the head of the pancreas, an enhancing mural nodule ≥ 5 mm, and a main pancreatic duct diameter ≥ 10 mm).

This classification was validated by a radiologist with 14 years of imaging experience.

### Fine-tuning of the model

**Fig. 2:**
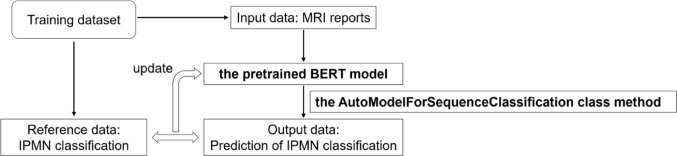
Fine-tuning process

We fine-tuned the pretrained Bidirectional Encoder Representations from the Transformers Japanese model (https://huggingface.co/cl-tohoku/bert-base-japanese) on a workstation equipped with a central processing unit of Core™ i9-12900F (Intel), a graphic processing unit of GeForce RTX 3090 (NVIDIA), and 128-GB RAM using the programming language of Python (version 3.10.13) (https://www.python.org/) and Transformers library (version 4.35.2) (https://huggingface.co/). The model comprised 12 layers, 768 dimensions of hidden states, and 12 attention heads. This model was pretrained using the Japanese Wikipedia as of September 1, 2019. Using the AutoModelForSequenceClassification class method, the model was fine-tuned to classify reports into five groups based on the logits for each group. Based on our previous experience, the number of epochs was set to 10, and the other hyperparameters were set to their default values. Because of the randomness of the training process, such as initialization of parameters and the shuffling of the training data, 15 sessions were performed on the training and validation datasets, and the model with the best performance was adopted. These processes are shown in Fig. 2.

### Test phase of the fine-tuned model and readers

The best-performing model on the validation dataset was evaluated on the test dataset. Two radiology residents (Readers 1 and 2 with 3 and 2 years of diagnostic imaging experience, respectively) and an intern (Reader 3 with 2 years of medical experience) manually sorted the reports in the test dataset into five groups, and their performance was compared with that of the model. Readers 1, 2, and 3 were blinded to the patient background information and performed the classification independently. Before this evaluation, a radiologist with 14 years of imaging experience randomized all the test datasets. The results and the time required to complete the classification were recorded.

### Statistical analyses

Statistical analyses were performed using R (version 4.4.2) (https://www.r-project.org/). McNemar analysis was performed to compare the sensitivity and accuracy of the fine-tuned LLM with those of the readers. Sensitivity for a given class was defined as the proportion of true positive cases correctly identified among all actual cases of that class. Receiver operating characteristic (ROC) curve analysis was performed to evaluate the performance of the fine-tuned LLM in differentiating groups 0–1 from groups 2–4, groups 0–2 from groups 3–4, and groups 0–3 from group 4 by calculating the area under the ROC curve (AUC). Kruskal–Wallis tests followed by post hoc Dunn’s test were performed to compare classification times between the LLM and each of readers 1, 2, and 3. Because multiple comparisons were performed for the AUC, sensitivities, and classification time, Bonferroni correction was applied, and statistical significance was set at a *p*-value of < 0.017 (= 0.050 / 3).

## Results

### Patients

Table 1 presents the dataset after the extraction. The training, validation, and test datasets contained 3,309, 159, and 290 reports, respectively, and no significant differences in mean age or sex distribution were observed in the respective datasets.

**Table 1:** Patient demographic data and distribution in each group

	Training	Validation	Test
Number of reports	3309	159	290
Age (mean ± standard deviation)	69.7 ± 10.3	69.1 ± 10.7	68.8 ± 10.6
Sex (male/female)	1527/1782	77/82	122/168
Number of reports in each group			
Group 1	261	11	23
Group 2	904	47	96
Group 3	1271	57	107
Group 4	522	20	40
Group 5	351	24	24

### Performance of the fine-tuned model on the validation dataset

Fifteen runs were performed on the training and validation datasets. Table 2 presents the accuracy of each model. One of the highest-performing models, Model 5, was adopted as the final model. Model 5 could classify the reports in the validation dataset with an accuracy of 0.981.

**Table 2:** Model’s accuracy on the validation dataset

Model	Accuracy	Model	Accuracy	Model	Accuracy
1	0.975	6	0.975	11	0.981
2	0.975	7	0.358	12	0.975
3	0.975	8	0.981	13	0.358
4	0.969	9	0.975	14	0.975
5	0.981	10	0.981	15	0.975

### Test phase of the fine-tuned model and readers

Table 3 presents the confusion matrix for the reference standard *versus* prediction data by the best fine-tuned LLM and readers. Table 4 presents the accuracy, sensitivity, and time required for categorizing the fine-tuned LLM and the three readers. The accuracy of the fine-tuned LLM on the test dataset was 0.966, which was comparable to those of Readers 1 and 2 (0.931–0.972) and significantly better than that of Reader 3 (0.907) (*p* = 0.003).

**Table 3:** Confusion matrix for the reference standard and prediction data

	Reference standard				
	Group 0	Group 1	Group 2	Group 3	Group 4
	(*n* = 23)	(*n* = 96)	(*n* = 107)	(*n* = 40)	(*n* = 24)
Large language model					
Group 0	22	0	1	1	0
Group 1	0	94	0	0	0
Group 2	0	1	104	0	2
Group 3	1	0	2	39	1
Group 4	0	1	0	0	21
Reader 1					
Group 0	21	1	0	0	0
Group 1	1	95	0	0	1
Group 2	0	0	103	0	0
Group 3	0	0	2	40	0
Group 4	1	0	2	0	23
Reader 2					
Group 0	11	0	1	0	0
Group 1	11	96	1	0	1
Group 2	0	0	103	0	1
Group 3	1	0	2	40	2
Group 4	0	0	0	0	20
Reader 3					
Group 0	22	5	11	5	2
Group 1	1	91	1	0	0
Group 2	0	0	93	0	0
Group 3	0	0	2	35	0
Group 4	0	0	0	0	22

**Table 4:** Performance of the fine-tuned LLM and readers

	Fine-tuned LLM	Reader 1		Reader 2		Reader3	
		Score	Comparison	Score	Comparison	Score	Comparison
Accuracy	0.966 (0.938–0.983)	0.972 (0.946–0.988)	0.752	0.931 (0.895–0.957)	0.055	0.907 (0.867–0.938)	0.003
Sensitivity							
Group 0	0.957 (0.781–0.999)	0.913 (0.720–0.989)	1	0.478 (0.268–0.694)	0.003	0.957 (0.781–0.999)	1
Group 1	0.979 (0.927–0.997)	0.99 (0.943–1.000)	1	1 (0.944–1)	N/A	0.948 (0.883–0.983)	0.371
Group 2	0.972 (0.920–0.994)	0.963 (0.907–0.990)	1	0.963 (0.907–0.990)	1	0.869 (0.790–0.927)	0.006
Group 3	0.975 (0.868–0.999)	1 (0.871–1)	N/A	1 (0.871–1)	N/A	0.875 (0.732–0.958)	0.221
Group 4	0.875 (0.676–0.973)	0.958 (0.789–0.999)	0.617	0.833 (0.626–0.953)	1	0.917 (0.730–0.990)	1
Time required(s)	25	1887		2122		2646	

Values in parentheses indicate 95% confidence intervals

*N/A* not applicable

**Fig. 3:**
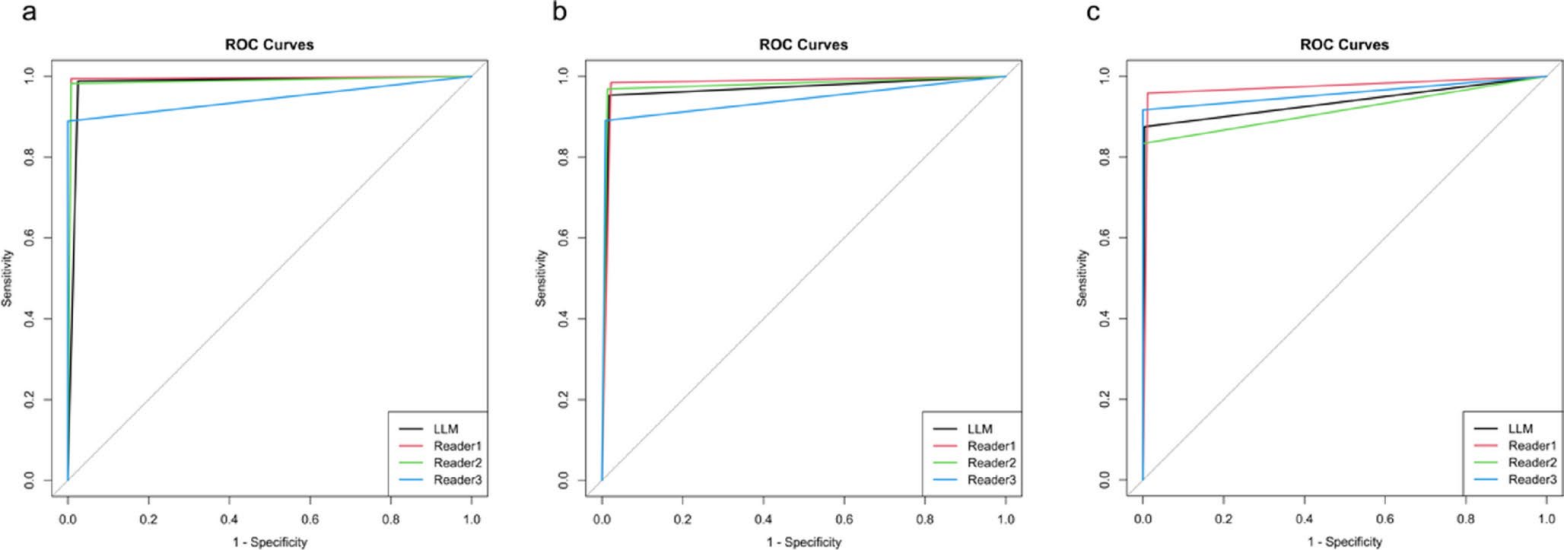
Receiver operating characteristic (ROC) analysis was performed to evaluate the performance of the fine-tuned LLM by calculating the area under the ROC curve (AUC). a: ROC curves distinguishing Groups 0 and 1 from Groups 2–4 (cyst diameter ≥ 10 mm). The AUCs for the fine-tuned LLM, Reader 1, Reader 2, and Reader 3 were 0.982 [0.965–0.998], 0.993 [0.983–1.000] (*p* = 0.146), 0.987 [0.974–1.000] (*p* = 0.535), and 0.944 [0.921–0.968] (*p* = 0.012), respectively. b: ROC curves distinguishing Groups 0–2 from Groups 3 and 4 (cyst diameter ≥ 20 mm). The AUCs for the fine-tuned LLM, Reader 1, Reader 2, and Reader 3 were 0.968 [0.940–0.995], 0.981 [0.963–0.999] (*p* = 0.404), 0.978 [0.955–1.000] (*p* = 0.467), and 0.941 [0.902–0.980] (*p* = 0.226), respectively. c: ROC curves distinguishing Groups 0–3 from Group 4 (WF and HRS). The AUCs for the fine-tuned LLM, Reader 1, Reader 2, and Reader 3 were 0.936 [0.868–1.000], 0.974 [0.932–1.000] (*p* = 0.365), 0.917 [0.841–0.993] (*p* = 0.689), and 0.958 [0.902–1.000] (*p* = 0.535), respectively, for WF and HRS

The sensitivity of the fine-tuned LLM was significantly better than that of Reader 2 in Group 0. Furthermore, the sensitivity of the fine-tuned LLM (0.972–0.979) tended to be superior to that of Reader 3 (0.869–0.948) in Groups 1–3, and a significant difference in sensitivity was observed in Group 2 (0.972 vs. 0.869) (*p* = 0.006). Figure 3 presents the ROC curves for the classification performance of the fine-tuned LLM and readers. The AUC of the fine-tuned LLM was 0.982 for distinguishing Groups 2–4 from Groups 0 and 1, and the fine-tuned LLM was significantly superior to Reader 3 (0.944) (*p *= 0.012). For distinguishing Groups 3 and 4 from Groups 0–2, no significant differences in AUC were observed between the fine-tuned LLM and readers.

The fine-tuned LLM completed the test dataset within 25 s, which was approximately 75 times faster than Reader 1, 85 times faster than Reader 2, and 106 times faster than Reader 3 (*p* < 0.001 for all).

## Discussion

In this study, we fine-tuned an LLM to classify abdominal MRI reports based on the Revised International Consensus Fukuoka Guidelines [5]. Our results revealed that the fine-tuned LLM could classify MRI reports of BD-IPMNs with high sensitivity and accuracy, which were comparable to or significantly better than those of less experienced human readers. Furthermore, the time required to complete the test dataset was remarkably shortened.

Among the readers, Reader 3, an intern, demonstrated the lowest accuracy, which was significantly lower than that of the fine-tuned LLM. Furthermore, Reader 3 had the longest turnaround time, and Reader 1, who had a longer diagnostic imaging experience, had a shorter turnaround time than Reader 2. This difference may reflect the experience with diagnostic imaging and familiarity with BD-IPMN classification. The number of IPMN cases is increasing because of the widespread use of imaging tests and the aging population [20]. Radiology residents at any institution will be required to make IPMN reports according to the guidelines, and the fine-tuned LLM developed in this study will be of great help for them in their daily practice. Furthermore, the fine-tuned LLM made it possible to classify patients with high accuracy and in a short time, which will be very useful for selecting subjects in future IPMN studies.

The sensitivity of the fine-tuned LLM was lower in classifying Group 4 than in classifying the other groups. This phenomenon may be attributed to the fact that the WF and HRS included various evaluation items, such as the main pancreatic duct diameter, walled nodules, and cyst diameter, and that the number of Group 4 reports was fewer than that of reports of the other groups. In the test dataset, the LLM was incorrect in 3 of the 24 Group 4 cases. Among the wrong cases, the LLM could not capture the growth rate and judged only based on the cyst diameter in two cases, and in the remaining case, the LLM was unable to capture nodules smaller than 5 mm with contrast enhancement and judged only based on the cyst diameter. The training dataset contained only four cases in which only the augmentation rate corresponded to WF and only one case in which only the nodule with contrast enhancement corresponded to WF, which would have been the reasons for the relatively lower sensitivity of the LLM in classifying Group 4. Improvements could be achieved by including more Group 4 reports and by including reports that meet the various conditions of WF and HRS.

This study has some limitations that should be considered. First, the experiment was performed at a single institution and has not been validated for applicability to other institutions or other reporting systems. However, we have attempted to split the dataset into the training, validation, and test datasets in chronological order. According to Walston et al., although random splitting and cross-validation are categorized as internal datasets, temporal or geographical sets are categorized as external datasets [21]. Second, this model could not refer to previous reports. Reports stating “no significant change since last time” had to be included in Group 0, regardless of the cyst diameter. Furthermore, WF included the rate of cyst growth, which is difficult to determine from a single report. Third, the clinical utility of LLMs has been widely reported. Although this study focused solely on radiology reports, models have also been developed to classify early and advanced stages of pancreatic ductal adenocarcinoma based on computed tomography findings, as well as to differentiate between pancreatic adenosquamous carcinoma and pancreatic ductal adenocarcinoma [22, 23]. Fourth, the developed model was based on radiology reports rather than on images. While image-based intelligent classification may offer high clinical value, this remains a subject for future research.

In conclusion, this study revealed that the fine-tuned LLM can classify BD-IPMNs based on the Revised International Consensus Fukuoka Guidelines with performance comparable to or significantly better than that of less experienced human readers and remarkably reduced the time required for categorizing.

## Electronic supplementary material

Below is the link to the electronic supplementary material.

Supplementary Material 1

## Data Availability

No datasets were generated or analysed during the current study.
