# Supplementary material for: Efficacy of a large language model in classifying branch-duct intraductal papillary mucinous neoplasms
Source: Abdom Radiol (NY). 2025 Jun 11;51(1):417–23. doi: 10.1007/s00261-025-05062-z (PMC12830448; doi:10.1007/s00261-025-05062-z)
Supplement: Supplementary file 1 — Supplementary Material 1 [file 261_2025_5062_MOESM1_ESM.pptx]

## Slide 1
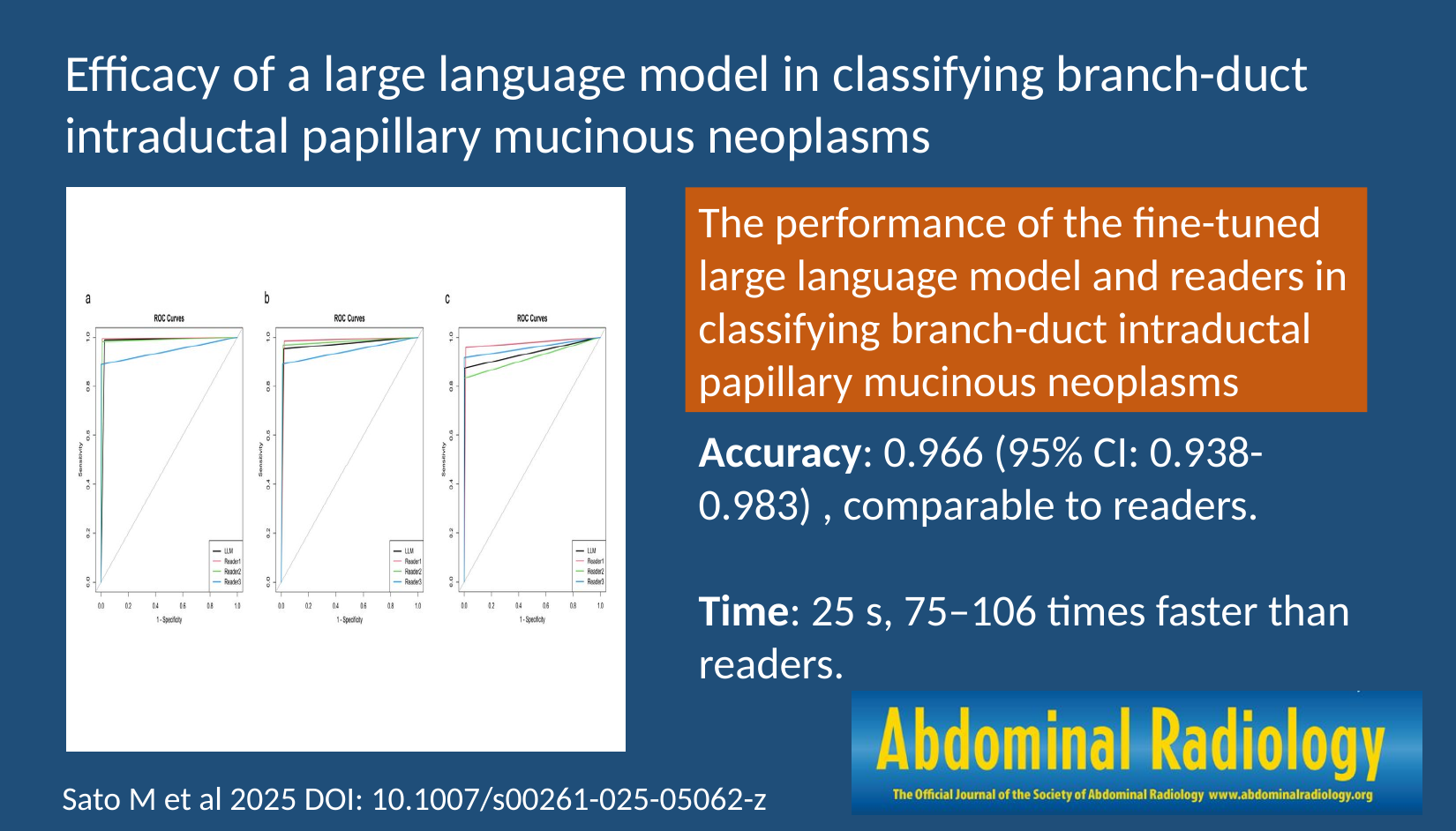

Efficacy of a large language model in classifying branch-duct intraductal papillary mucinous neoplasms
The performance of the fine-tuned large language model and readers in classifying branch-duct intraductal papillary mucinous neoplasms
Accuracy: 0.966 (95% CI: 0.938-0.983) , comparable to readers.
Time: 25 s, 75–106 times faster than readers.
Graph / figure / visual illustrating key point of the paper
Sato M et al 2025 DOI: 10.1007/s00261-025-05062-z
